# Molecular conservation of estrogen-response associated with cell cycle regulation, hormonal carcinogenesis and cancer in zebrafish and human cancer cell lines

**DOI:** 10.1186/1755-8794-4-41

**Published:** 2011-05-16

**Authors:** Siew Hong Lam, Serene GP Lee, Chin Y Lin, Jane S Thomsen, Pan Y Fu, Karuturi RK Murthy, Haixia Li, Kunde R Govindarajan, Lin CH Nick, Guillaume Bourque, Zhiyuan Gong, Thomas Lufkin, Edison T Liu, Sinnakaruppan Mathavan

**Affiliations:** 1 Genome Institute of Singapore, #02-01Genome, 60 Biopolis Street, 138672, Singapore; 2 Department of Biological Sciences, National University of Singapore, 14 Science Drive 4, 117543, Singapore; 3 Department of Microbiology and Molecular Biology, Brigham Young University, 753 WIDB, Provo, UT 84602, USA; 4 NUS Environmental Research Institute (NERI), TL #02-02, Engineering Drive 1, 117411, Singapore; 5 Center for Nuclear Receptors and Cell Signaling, Department of Biology and Biochemistry, University of Houston, 3013 Science & Engineering Research Center, Houston, TX 77204, USA

**Keywords:** zebrafish, microarray, estrogen, anti-estrogen ICI 182,780, estrogen-responsive genes, signaling pathways, carcinogenesis, human cancer cell lines, molecular conservation, model organism

## Abstract

**Background:**

The zebrafish is recognized as a versatile cancer and drug screening model. However, it is not known whether the estrogen-responsive genes and signaling pathways that are involved in estrogen-dependent carcinogenesis and human cancer are operating in zebrafish. In order to determine the potential of zebrafish model for estrogen-related cancer research, we investigated the molecular conservation of estrogen responses operating in both zebrafish and human cancer cell lines.

**Methods:**

Microarray experiment was performed on zebrafish exposed to estrogen (17β-estradiol; a classified carcinogen) and an anti-estrogen (ICI 182,780). Zebrafish estrogen-responsive genes sensitive to both estrogen and anti-estrogen were identified and validated using real-time PCR. Human homolog mapping and knowledge-based data mining were performed on zebrafish estrogen responsive genes followed by estrogen receptor binding site analysis and comparative transcriptome analysis with estrogen-responsive human cancer cell lines (MCF7, T47D and Ishikawa).

**Results:**

Our transcriptome analysis captured multiple estrogen-responsive genes and signaling pathways that increased cell proliferation, promoted DNA damage and genome instability, and decreased tumor suppressing effects, suggesting a common mechanism for estrogen-induced carcinogenesis. Comparative analysis revealed a core set of conserved estrogen-responsive genes that demonstrate enrichment of estrogen receptor binding sites and cell cycle signaling pathways. Knowledge-based and network analysis led us to propose that the mechanism involving estrogen-activated estrogen receptor mediated down-regulation of human homolog *HES1 *followed by up-regulation cell cycle-related genes (human homologs *E2F4, CDK2, CCNA, CCNB, CCNE*), is highly conserved, and this mechanism may involve novel crosstalk with basal AHR. We also identified mitotic roles of polo-like kinase as a conserved signaling pathway with multiple entry points for estrogen regulation.

**Conclusion:**

The findings demonstrate the use of zebrafish for characterizing estrogen-like environmental carcinogens and anti-estrogen drug screening. From an evolutionary perspective, our findings suggest that estrogen regulation of cell cycle is perhaps one of the earliest forms of steroidal-receptor controlled cellular processes. Our study provides first evidence of molecular conservation of estrogen-responsiveness between zebrafish and human cancer cell lines, hence demonstrating the potential of zebrafish for estrogen-related cancer research.

## Background

Estrogen is known to be carcinogenic and there are several mechanisms postulated for its carcinogenic and tumor-promoting effects. One of the most widely acknowledged mechanism of estrogen carcinogenicity is the multiple estrogen-receptor signal-transduction pathways associated with increased cell proliferation and inhibition of apoptosis [1-3]. This could involve the direct genomic action of estrogen binding to nuclear estrogen receptors (ERα and/or ERβ), which then bind as dimers to estrogen-response elements (ERE) in the regulatory regions of estrogen-responsive genes in association with various basal transcription factors, coactivators, and corepressors to alter expression of genes involving in cell cycle control [1] and other tumor-promoting factors such as vascular endothelial growth factor [4]. Moreover, via non-genomic action, estrogen can also cause activation of protein kinases, including mitogen-activated protein kinases, and rapidly increases the levels of secondary messengers, such as cyclic AMP that can cross-talk with other growth factors (epidermal growth factor receptor and insulin-like growth factor 1 receptor) and signaling pathways, that are important in estrogen-dependent cell cycle regulation [2,3]. Another potential mechanism is via estrogen metabolism whereby oxidative metabolites of estrogen are shown to have genotoxic (formation of DNA adducts and oxidative DNA damage), mutagenic, transforming, and carcinogenic effects [5,6]. In addition, estrogen has been shown to cause over-expression of centrosome kinases (Aurora A and B) and centrosome amplification which can lead to chromosomal instability resulting in aneuploidy in early tumor foci that precipitates oncogenesis [7]. These evidences along with cancer epidemiological data of reproductive tissues had supported the classification of estrogen as a carcinogen.

The zebrafish is emerging as a cancer model that offers the high-throughput advantage of an in vitro model as well as the whole-animal physiology environment of an in vivo model [8]. The potential of zebrafish as a cancer model is derived from its strength as an experimental system for developmental biology and toxicology. Being a vertebrate, many of the developmental and physiological processes are conserved between zebrafish and mammals, from the anatomical level to the molecular level. Although zebrafish do not have certain organ-tissues or glands (e.g. mammary and prostate) found in mammals, similar molecules and signaling pathways involved in carcinogenesis may still be operating in human neoplasms. Hence, zebrafish is known to be susceptible to carcinogens affecting humans and develop a wide spectrum of cancers resembling human malignancies [8,9]. Moreover, the high amenability of zebrafish to various molecular techniques and genetic manipulation, coupled with a vast genome resources including a near complete genome sequence and gene expression platforms (e.g. microarray and RNA sequencing) has empowered zebrafish with versatility for various cancer research [10-12]. From inducing tumors driven by oncogenes in specific tissues and fluorescent imaging of tumorigenesis in living transgenic zebrafish to screening of chemical and genetic modifiers of cancer, the zebrafish model can be used for addressing basic tumor biology and high-throughput drug screening applications. The zebrafish has also been employed for toxicological characterization of environmental carcinogens and endocrine disruptors that could pose public health-risks [13,14].

As part of our research endeavor to explore the potential of using zebrafish for modeling estrogen-related cancer research (such as estrogen-induced carcinogenesis, estrogen-responsive cancer model and for screening of estrogen receptor modulators), we performed microarray experiments on zebrafish exposed to estrogen 17β-estradiol (E2) and, a combination of E2 and anti-estrogen ICI 182,780 (E2+ICI). Although zebrafish is known to be responsive to estrogen and contain estrogen receptors similar to human ERα and ERβ and their developmental expression patterns had been characterized [15-17], it has not been demonstrated whether the estrogen-responsiveness in terms of genes and signaling pathways are similar to those operating in estrogen-responsive human cancer cells. In this study, we first identified estrogen-responsive genes in zebrafish that were sensitive to both estrogen and anti-estrogen. By human homology mapping and knowledge-based data mining, we found that many of the genes were associated with cell cycle, DNA replication, DNA damage and repair and cancer. By comparing with estrogen-responsive human cancer cell lines, we identified a core set of conserved estrogen-responsive genes that have significant enrichment of ER binding sites as well as cell cycle signaling pathways. Further, network analysis reveals mechanistic insights into a conserved estrogen-mediated cell cycle regulation and signalling pathway. This study provides the first molecular evidence of conserved estrogen-responsiveness between zebrafish and human cancer cell lines, further demonstrating zebrafish's potential for estrogen-related cancer research.

## Methods

### Experimental design

Experimental procedures were performed within the guidelines of National University of Singapore's Institutional Animal Care and Use Committee. A batch of healthy adult males were exposed to the medium containing 17β-estradiol (E2; Sigma-Aldrich; 10 nM final concentration) and an equal number of males were exposed to the medium containing a combination of E2 (10 nM) and ICI 182,780 (Tocris Cookson) (1 μM final concentration). Control fishes (males) were maintained in water containing 0.01% (v/v) ethanol (ethanol control; ethanol was used to dissolve E2 and ICI). The final ethanol concentration in the control was similar to those in medium with E2 or E2+ICI. Four replicates were maintained for each treatment. Control and experimental animals were maintained in their respective medium for 96 hours and the medium was changed every day during the course of the experiment. Upon completion of the experiment at 96 hours, the fish were snapped frozen in liquid nitrogen and stored at -80°C for subsequent analysis.

### Experimental samples and RNA extraction

Total RNA was extracted from individual frozen fish belonging to control and experimental samples (ethanol control, E2 and E2+ICI treated). Frozen samples were homogenized to a crude powder form in a pre-cooled mortar and pestle. During the grinding, the sample was kept in frozen condition by adding liquid nitrogen to the sample in the mortar. Partially homogenized sample was transferred to a pre-cooled sterile graduated falcon tube containing appropriate amount of Trizol reagent (Invitrogen, USA) and homogenized completely using a motorized homogenizer. Total RNA was extracted from the samples using Trizol reagent according to manufacturer's instructions. Subsequently RNA was purified using Qiagen column and the quality was evaluated using gel electrophoresis. Reference RNA was prepared from equal amount of male and female total RNAs extracted from pooled liver from male and female fish, respectively. Sufficient amounts of reference RNA required for the entire project was prepared at one time and stored as 100 μl aliquots at -80°C.

### Zebrafish microarray and data processing

Compugen microarray set (Compugen, USA) containing 16,416 oligonucleotide probes representing zebrafish genes was used in this study. Briefly, the oligonucleotide probes were spotted onto poly-L-lysine coated microscope slides using a custom-built DNA microarrayer and post-processed following the standard procedures previously described [18]. Sample and reference RNA were reverse transcribed in the presence of Cy3-dUTP and Cy5-dUTP (Amersham Inc.), respectively, to fluorescently label the target cDNAs. The arrays were hybridized following the strategies described in [52]. A minimum of three good hybridizations were selected for the analysis. The signal intensities of Cy5 and Cy3 dyes in each spot and the local background were measured using the GenePix 4000B microarray scanner (Axon Instruments, USA) to calculate the net intensity of each spot for analysis. Microarray data from GenePix image analysis software (i.e gpr files) were subjected to Lowess normalization. There were 4 control (ethanol controls) samples, 3 samples treated with E2 and 3 samples treated with E2+ICI in the normalized data, respectively. The good arrays were selected based on scatter plot analysis.

Significance Analysis of Microarrays (SAM) was used to identify the prominent signals for the two kinds of treatments as previously described [19]. To boost the power of the test, we applied 3-class SAM on the whole array by excluding spots with more than 6 missing values. As a result, 1610 out of 16416 genes were selected at q-value < 8%. Subsequently, estrogen-responsive genes were identified by analyzing the expression values for the samples treated with E2 and E2+ICI with respect to control samples using 2-class SAM. The genes selected following this analysis displayed between 2- to 130-fold differential expressions. A cluster of 715 up-regulated genes were identified which showed significant up-regulation in E2. These genes displayed decreased expression both in the Control and E2+ICI compared to their level of expression in E2. Similarly, another cluster of 376 genes (down-regulated group) were identified which displayed significantly reduced expression in E2 compared to control and E2+ICI. Hence, the genes selected by the above analysis are estrogen-responsive and are sensitive to both E2 and ICI. Datasets extracted using the above statistical analysis were clustered and visualized as previously described [19]. The array data has been submitted to Gene Expression Omnibus database and the accession number is GSE27707.

### Quantitative Real-time PCR (qRT-PCR)

Transcription dynamics observed in the microarray experiments was validated using quantitative real time PCR (qRT-PCR) for a selected subset of up and down-regulated genes. The GeneBank Accession and primers used for qRT-PCR are given in the Additional File 1. Gene specific primers were designed using Primer Express software (version 3.0; Applied Biosystems). The strategy was to select two exons with a large intronic junction, and then the primer was designed across the junction to ensure the amplification is from the cDNA and not from genomic DNA. Total RNA extracted from the experimental samples were treated with RNAse free DNAse (Ambion, Austin, USA) to eliminate the contamination of genomic DNA and about 1ug of RNA was reverse-transcribed into cDNA using High-Capacity cDNA Reverse Transcription Kit (using random primers; Applied Biosystems). ABI system 7900HT Fast Real-Time PCR machine with 384 well formats was used for the analysis. Fluorescent nucleic acid dye SYBR green I (Applied Biosystems) was used for detection. Samples were tested in quadruplicates. The reaction mix without template served as negative control and beta actin served as positive control. The results were analyzed using Sequence Detection Software (version 2.3) and SDS-RQ manager (version 1.2; Applied Biosystems).

### Mapping of zebrafish estrogen responsive genes to human homologues

Most of the functional group and pathways enrichment analysis software use human UniGene Ids as one of the input source data. For comparative purpose between zebrafish and human cancer cell lines, we had to identify human homolog for the zebrafish estrogen-responsive genes. The zebrafish estrogen-responsive genes with GenBank accession numbers were clustered into UniGenes http://www.ncbi.nlm.nih.gov/unigene/ and these UniGenes were mapped to the HomoloGene database http://www.ncbi.nlm.nih.gov/entrez/query.fcgi?CMD=search&DB=homologene; Human UniGene build 200; HomoloGene Build 56) to obtain their human homologs. We have created a web-based tool http://123.136.65.67/ to map the zebrafish UniGene Ids to human HomoloGene [10]. This facilitate large-scale mapping of zebrafish estrogen-responsive genes to the corresponding human homologs.

### Ingenuity Pathway Analysis (IPA)

Pathways are graphical representations of molecular relationships between selected set of biological entities derived from diverse sources of established information. It is usually made up of nodes and edges where nodes represent biological entities (e.g. genes, proteins, complexes) and edges represent interactions (e.g. induction, inhibition, binding, regulation, phosphorylation etc) between nodes in the pathway. It presents an illustration of a 'focused' view of a biological function or a 'global' view of complex networks that are enriched in large scale microarray data sets. Functional properties and pathway enrichment of the estrogen-responsive genes of zebrafish were generated using Ingenuity Pathways Analysis (IPA; Version 6) (Ingenuity^® ^Systems, http://www.ingenuity.com). Information in this large database were obtained through manual curation peer-reviewed literature and continually updated with information such as modeled relationships between biological entities (genes, proteins, cells, tissues, etc.), canonical pathways and functional categories ("molecular and cellular functions", "disease and disorders" and "physiological system development and functions"). IPA will associate genes from the input dataset for different functional categories and calculate a p-value using the right-tailed Fisher's exact test to assess the statistical significance of the enriched genes for a functional category in relation to the initial input dataset and the total number of genes in the database involved in the function. A p-value < 0.05 indicates statistical significance or non-random association.

### Estrogen responsive element (ERE) and estrogen receptor(ER) binding site analysis

Human homolog of zebrafish estrogen-responsive genes (475 genes) were intersected with estrogen-responsive genes from 4 estrogen-responsive human cancer cell lines [T47D cells [20], MCF7 cells [21] and, MCF7 and Ishikawa cells (Thomsen et al. unpublished data); Additional Files 2, 3, 4, 5]. The human cell line datasets contained the following number of estrogen-responsive genes and were represented by UniGene identifiers: MCF7-1485 genes, T47D-975 genes, Ishikawa-1643 genes and MCF7-1531 genes. The randomization was done using all the human homolog of zebrafish (8056 human UniGene identifiers) generated by mapping all the zebrafish genes in the microarray to HomoloGene database as described earlier. The mapping location of each of the 139 conserved estrogen-responsive genes were retrieved using the University of California Santa Cruz (UCSC) Genome Browser (http://genome.ucsc.edu; May 2004 assembly from the database 'KnownGene and all_mrna'). Of the 139 genes, 133 were could be mapped using the UCSC genome browser. We scanned for scanning ERE motif along 20 kb sequences from the upstream of 5', downstream of 3' and within the gene of the estrogen-responsive genes of zebrafish and scanned for the motif. We also scanned for the ERE motif in the whole genome of zebrafish and analyzed the possible enrichment of ERE motif in the estrogen-responsive genes compared to the whole genome distribution of the motif. In addition, the genes were then scanned for ER-binding site in the neighbourhood (100kbp range) using a combined list of ER-binding sites. The combined list was derived from All-ER binding data for MCF7 cell [21,22], Fun et al 2010 in preparation]. The Binomial test were used to determine the statistical significance of the intersection between datasets as well as the ERE motif and ER-binding site analyses. A p-value < 0.05 indicates statistical significance.

## Results and Discussion

### Identification of zebrafish estrogen-responsive genes via whole-organism transcriptome profiling and qRT-PCR validation

**Figure 1:**
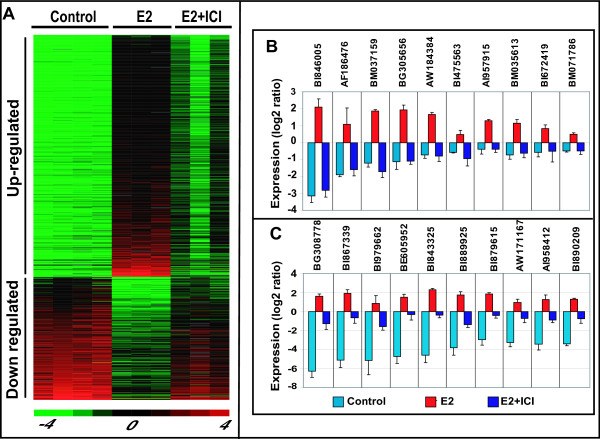
**Expression profile of zebrafish estrogen-responsive genes**. **A**. Total of 1092 estrogen-responsive genes (715 up-regulated and 377 down-regulated) following E2 treatment which are also partially suppressed or normalized in E2+ICI treatment. **B**. Selected estrogen-responsive genes having their estrogen-induced transcriptional changes almost completely suppressed or normalized by E2+ICI treatment. **C**. Selected estrogen-responsive genes having their estrogen-induced transcriptional changes partially suppressed by E2+ICI treatment.

Estrogen-responsive genes were determined by comparing whole transcriptome profiles of male zebrafish treated with vehicle (control group), E2 and E2+ICI using SAM analysis (Significant Analysis of Microarrays; see [19]). We anticipated that estrogen-responsive genes would be deregulated in response to E2 treatment and the level of deregulation would be partially suppressed or normalized when the anti-estrogen drug ICI is included in the E2 treatment since ICI is known to bind estrogen receptor monomers and prevent dimerization, hence blocking estrogen receptor signalling to targeted genes [23]. Indeed, SAM analysis of zebrafish whole-organism transcriptome profiles identified a total of 1092 estrogen-responsive genes (715 up-regulated and 377 down-regulated) following E2 treatment which were also partially suppressed or normalized in E2+ICI treatment (Figure 1A; Additional File 6 and 7). A closer examination of the transcriptome profiles (Figure 1B and 1C) revealed changes of transcript abundance from about 2-fold to 128-fold following E2 treatment. In a subset of responsive genes, E2+ICI treatment almost completely suppressed or normalized the estrogen-induced transcriptional changes (Figure 1B), while in another sub-set of genes E2+ICI treatment partially suppressed the E2 induced transcriptional changes (Figure 1C). These observations indicate that our microarray experiment successfully captured genes responding (to varying levels) specifically to estrogen and the anti-estrogen treatment.

**Figure 2:**
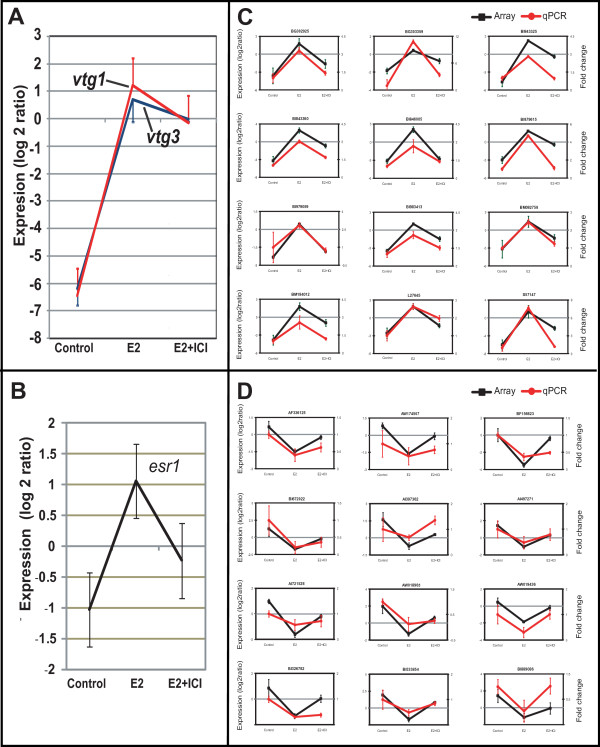
**Validation of microarray data by qRT-PCR**. **A**. Expression levels of *vtg1 *and *vtg3 *and **B**. *esr1*. **C**. Selection of representative up-regulated genes. **D**. Selection of representative down-regulated genes. The data represents average of 3 replicates and presented as mean and standard deviation.

To validate the reliability of the microarray data, we performed qRT-PCR on vitellogenins (*vtg1, vtg3*) and zebrafish homolog of estrogen receptor alpha (*esr1*), which are well known biomarker genes responsive to estrogen[24,25]. Expression of *vtg1 *and *vtg3 *was induced almost 400-fold and 200-fold, respectively, following E2 treatment when compared to the control group but was only induced to approximately 80-fold in E2+ICI treatment (Figure. 2A).This indicate that E2-induced *vtg *expression was suppressed approximately 2.5 to 5 fold by the addition of ICI [fold-change above reference RNA (Log_2_ratio)]. As for *esr1*, E2 treatment increased the level of expression by about 4-fold and the inclusion of ICI suppressed the transcript level by about half (Figure. 2B). In addition to the known targeted biomarker genes, we have validated a representative set of up-regulated and down-regulated genes (Figure 2C and 2D; primer sequence in Additional File 1) which further confirmed the estrogen-responsiveness of the genes and the reliability of the microarray data. To the best of our knowledge, this is the first comprehensive study to identify the estrogen-responsive genes in a whole organism level involving treatments with estrogen and anti-estrogen in combination.

### Human homolog mapping and identification of putative novel estrogen-responsive genes in zebrafish

Genes identified in this study were first categorized based on their original annotations such as functionally known genes, different groups of Expressed Sequence Tags (ESTs) and ZGC clones (NIH Zebrafish Gene Collection, full-length sequenced clones without any known function). Of the 1092 zebrafish estrogen-responsive genes, about 31.5% were fully annotated while the rest were ESTs (49%) or ZGC (19.5%) clones (Additional File 8). The estrogen-responsive genes were further mapped to NCBI HomoloGene database (HomoloGene build 56 and Human UniGene build 200) and 475 (43.5%) of the estrogen-responsive genes have corresponding human homologs (Additional File 9). A subset of zebrafish full-length sequenced genes (ZGC clones) with no functional annotation that are sensitive to E2 and antiestrogen (Additional File 10) were also identified for the first time. A search in the NCBI UniGene database linked to the GenBank IDs of these ZGC clones indicated that many have ESTs that are expressed in the zebrafish reproductive system (based on tissue specific ESTs) and some of these clones have moderate homology to human proteins. Hence, we have identified a number of putative novel estrogen-responsive genes in zebrafish; of these, some may have human homolog while the remainder appear to be fish specific. Characterization of some of the new target genes might provide additional insights into both general and species-specific ER regulated gene expression.

### Identification of zebrafish estrogen-responsive genes associated with carcinogenesis or cancer

**Figure 3:**
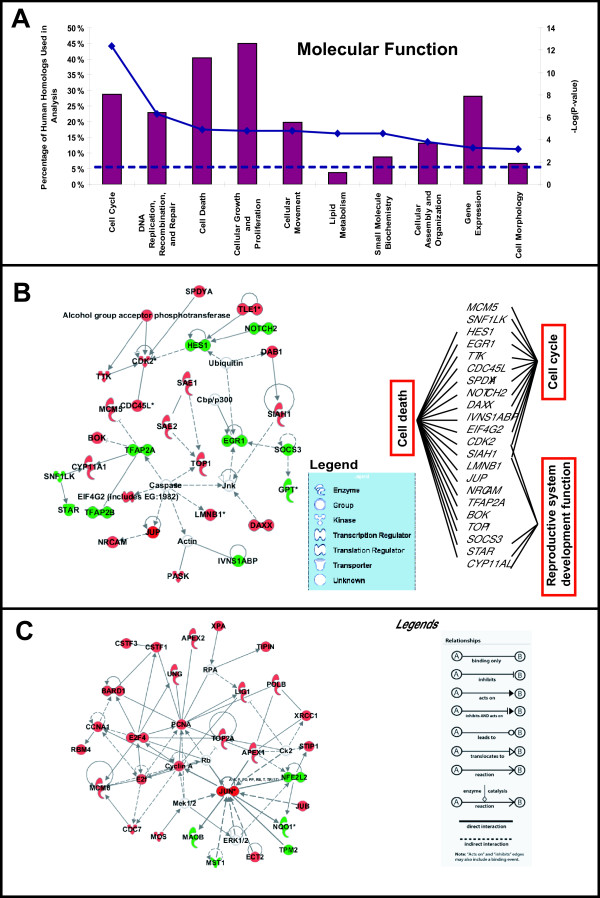
**Knowledge-based functional and network analyses of human homologs of zebrafish estrogen-responsive genes**. **A**. Selected top molecular and cellular functions significantly (P-value < 0.05) associated human homologs of zebrafish estrogen-responsive genes. Histograms are read with reference to 'Percentage of Human Homologs Used in Analysis' axis while solid and dashed lines are read with reference to '-Log P-value' axis. 'Percentage of Human Homologs Used in Analysis' refers to the percentage of the total 289 human homologs used in the analysis. Solid line represents the inverse logarithm (base 10) of the p-value for each group of biological association [greater -Log (P-value) correlates with greater statistical significance] while the dashed-line represents the significant threshold where the p-value = 0.05. **B**. Top most significant network assembled de novo using 30 focus molecules associated with cell cycle, cell death and reproductive system development and function. The inset shows the molecules in the network that are involved in more than one function. **C**. Second top significant network assembled de novo from 30 focus molecules associated with DNA replication, recombination and repair, cell cycle and cancer. Red nodes represent genes encoding respective molecules are up-regulated in response to estrogen and green nodes represent down-regulation. Interactions are represented by edges connecting 2 nodes. Different types of edges represent the direction and effect of interactions as given in the legends.

To obtain biological insights into the zebrafish estrogen-responsive genes, we analyzed the human homologs of zebrafish estrogen-responsive genes using knowledge-based pathway data mining and network generator algorithm [Ingenuity Pathways Analysis; IPA]. Of the 475 human homologs of E2 responsive genes identified in zebrafish, 325 were eligible for network analysis while 289 were eligible for function/pathway analysis. Genes that were not eligible for network/function/pathway analysis were genes with no reported involvement, hence not assigned, in any known molecular function/pathway in the database. The algorithm calculates the significance of an association (p-value < 0.05 indicate non-random association) using the right-tailed Fisher's exact test based on the number of genes from the 289 human homologs that are associated in a given molecular function/pathway, relative to the total number of genes found in the molecular function/pathway in IPA. The analysis revealed that the top five significant molecular functions were: i) Cell Cycle, ii) DNA Replication, Recombination, and Repair, iii) Cell Death, iv) Cellular Growth and Proliferation, and v) Cellular Movement, and they involved 20-45% of the 289 homologs (p-value = 4.21E-13 to 2.58E-02; Figure 3A andAdditional File 11). Given the strong association with cell cycle processes, it is not surprising that the analysis also revealed that approximately 42% of the homologs were significantly (p-value = 1.13E-8 to 2.32E-02; Additional File 12) associated with cancer ranging from tumorigenesis, neoplasia, to various cancer types including those of the reproductive system (uterine, endometrial, breast, ovarian and prostate).

Next, to discover and visualize the biological connectivity of the estrogen-responsive genes, the IPA network generating algorithm is used to maximize the interconnectedness of the genes based on all known connectivity in the database. The algorithm also incorporates other genes from the database to maximize the connectivity with the estrogen-responsive genes to assemble a 'focus gene network' and it handles a maximum of 35 focus genes per network. A network score is generated based on the hypergeometric distribution and is calculated with the right-tailed Fisher's exact test. The top 5 scoring networks were assembled de novo using 22-29 focus genes with associated functions ranging from cell cycle, cancer, DNA replication, recombination and repair to gene expression, dermatological diseases, and reproductive system development and function (Additional File 13). Interestingly, the top most significant (p-value = 1.00E-46) gene network was assembled with 29 focus genes involved in cell cycle, cell death, and reproductive system development and function (Figure 3B), hence recapturing genes that are involved in the classical estrogen-induced cellular proliferative effects in reproductive tissues. In this network we observed that several transcription regulators such as human homologs *DAXX, HES1, EGR1, TFAP2A, TFAP2B *and *NOTCH2 *that are involved in cell death were deregulated while interconnected *CDC45L, CDK2, EIF4G2, MCM5, SAE1, SPDYA, TOP1 *and *TTK *involved in cell cycle progression and division were up-regulated, signifying that the cell cycle molecular machinery has been activated by estrogen. This is further supported by the second top significant network (Figure 3C; p-value = 1.00E-43) which is assembled from 28 focus genes known to be associated with DNA replication, recombination and repair, cell cycle and cancer. In this network, human homologs *CCNA1, JUN, E2F4 *and *PCNA *formed highly connected network hubs that are up-regulated to control many of the cell cycle progression and DNA replication processes. In contrast, human homologs *NFE2L2, NQO1 *and *MAOB *which are critical to protect cells from oxidative stress damage were down-regulated and this could lead to accumulation of oxygen radicals that can cause cellular damage including DNA. Estrogen metabolism itself has been reported to be genotoxic and can cause oxidative DNA damage [5,26]. Hence, genes associated with DNA damage and repair such as human homologs *APEX1, APEX2, XPA, XRCC1 *and *PCNA*, were up-regulated presumably to repair oxidative DNA damages resulted from estrogen metabolism and/or replication errors due to increased DNA replication activity. Moreover, increased expression of human homolog *JUB *as captured in the network could lead to increase activity of the up-regulated *JUN *[27] which is an estrogen-responsive proto-oncogene known for its malignant transforming properties and tumorigenic potentials [28,29]. Likewise, up-regulation of *JUB *could also increase *AURKA *activity[30], a known estrogen-responsive centrosome kinase, that could induce spindle defects, chromosome mis-segregation, and genomic instability leading to neoplastic transformation [7,31].

Taken together, the analysis revealed that treatment of estrogen in zebrafish could induce a large number of estrogen-responsive genes involved in cell cycle, proliferation, DNA replication, DNA damage and repair, recapitulating known mechanisms that are associated with estrogen-dependent carcinogenesis and/or cancer. The responsiveness to ICI 182,780 also suggests its potential for screening of anti-estrogens and selective estrogen receptor modulators (SERMs). The findings further lead us to identify conserved estrogen-responsive genes between zebrafish and human cancer cell lines.

### Conservation of estrogen-responsive genes between zebrafish and human cancer cells

**Figure 4:**
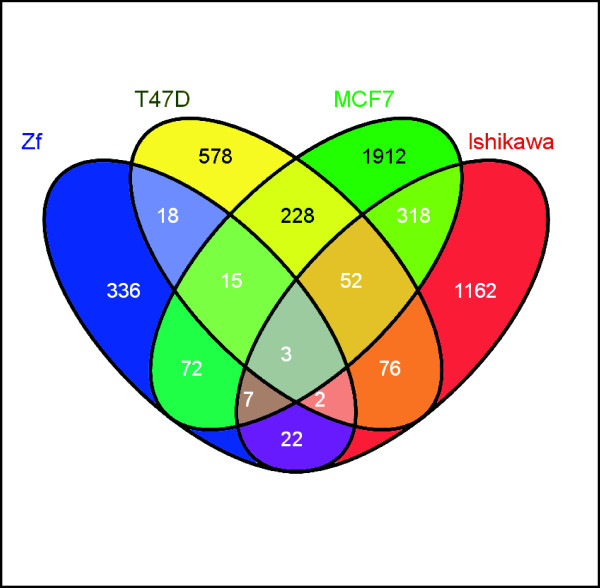
**Overlap between estrogen responsive genes of zebrafish and human cancer cells**: Estrogen responsive genes identified in zebrafish and human cancer cells (T47D [20]; MCF7, [21], Ishikawa and MCF7, Thomsen et al, 2008, unpublished) were compared and the number of overlapping genes between the datasets is presented. Data for MCF7 cells from Carroll et al. [21] and Thomsen (2008 unpublished) were pooled together for this analysis.

To identify estrogen-responsive genes that are conserved between zebrafish and estrogen-responsive human cancer cell lines, genes responding to estrogen treatment in zebrafish (current study) and in human cancer cells [T47D cells [20], MCF7 cells [21] and, MCF7 and Ishikawa cells (Thomsen et al. unpublished data; Additional Files 2, 3, 4, 5) were compared. Of the 475 human homologs of zebrafish estrogen-responsive genes, 139 (29%) were responsive to estrogen in one or more human cancer cell lines (Figure 4; Additional File 14). The percentage intersection of the human homologs of zebrafish estrogen-responsive genes (Additional File 15 ranged from 7% (zebrafish and Ishikawa cells) to 14% (zebrafish and MCF7_1 cells). Between the human cancer cell lines, the percentage intersection ranged from 8% (MCF7_1 and Ishikawa cells) to 26% (MCF7-1 and MCF7-2 cells; Additional File 16). Hence, the percentage intersection between zebrafish and human cancer cell line datasets were more than half of the percentage intersection among the human cancer cell lines. Although the intersections were not high, this is not unexpected considering the differences in experimental designs, cell lines, reagents, platforms, data processing and analysis approaches that would have generated variations in capturing the molecular signals in response to estrogen. Furthermore, differences in the estrogen-responsiveness of genes captured at the whole-organism level of a fish and human cancer cell lines were expected. Despite these differences, we were able to identify a core set of 139 conserved estrogen-responsive genes that are active in zebrafish and human cancer cell lines. This also suggests that the core set of 139 conserved estrogen-responsive genes are highly robust with regard to their responsiveness to estrogen since they were able to be identified as 'significantly deregulated' despite the biological, technical and experimental variations.

### Enrichment analysis of ERE motif and ER binding sites in conserved estrogen-responsive genes

To examine potential ER-mediated transcriptional regulation of the 139 conserved estrogen-responsive genes, we analyzed the distribution of ERE motifs (ERE: GGTCAnnnTGACC) in their flanking regions. We scanned the 20 kb flanking regions of the 139 conserved estrogen-responsive genes and the whole genome of zebrafish for the distribution of EREs. We found that, the ERE motifs are randomly distributed in the zebrafish genome and not enriched in the regulatory regions of zebrafish estrogen-responsive genes (Additional File 17). This observation is similar to the data reported for the distribution of ERE in the human genome and human estrogen-responsive genes [32]. This further suggests that presence ERE motifs are not correlated with estrogen-responsiveness of genes throughout vertebrate.

Next, we scanned for ER binding sites in the cis-regulatory regions (within 100 kb) adjacent to these 139 conserved estrogen-responsive genes by mapping the human genes to a list of known (experimentally validated by ChIP-chip and ChIP-seq analysis) ER binding sites ([20,21] (Fun et al 2010; personal communication)] found in the human genome. Our analysis showed that out of the 139 conserved estrogen-responsive genes, 65 had ER binding sites (Additional File 14). Moreover, a total of 158 ER binding sites were significantly enriched compared to an expected 65 binding sites (Binomial p-value < 2.2E-16; Additional File 17). In addition, we also scanned for ER binding sites for the rest 336 'non-conserved' human homologs of zebrafish estrogen-responsive genes that did not overlap with any of the human cancer cell lines. The 336 'non-conserved' genes had a total of 210 ER binding sites. Therefore, on an average, the number of binding site per gene is calculated to be 1.16 for the conserved genes and 0.62 for the non-conserved genes. Thus the current analysis suggests that ER binding site is enriched in conserved estrogen-responsive genes compared to the 'non-conserved' human homologs of zebrafish estrogen-responsive genes that did not overlap with any of the human cancer cell lines. The findings also indicate that comparative transcriptomics approach between two phylogenetically distant species could help to narrow down estrogen-responsive genes enriched with ER binding sites. The set of genes with experimentally validated ER binding site would suggest that these are direct ER-regulated genes. Estrogen-responsive genes without ER binding sites may be regulated through interaction with other transcription factors or via non-genomic actions.

### Conserved estrogen-responsive signaling pathways and novel insights

**Figure 5:**
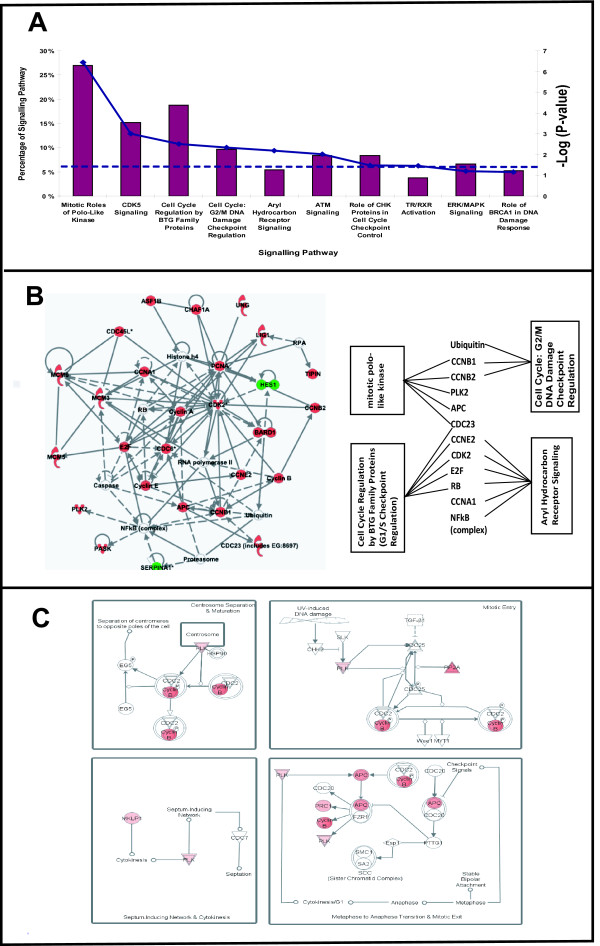
**Knowledge-based signalling pathway and network analyses of conserved estrogen-responsive genes between zebrafish and human cancer cell lines**. **A**. Selected top signaling pathways significantly (P-value < 0.05) associated with conserved estrogen responsive genes. Histograms are read with reference to 'Percentage of Signalling Pathway' axis while solid and dashed lines are read with reference to '-Log P-value' axis as in Figure 3A. 'Percentage of Signalling Pathway' refers to the percentage of the total molecules in each respective signalling pathway. **B**. Top most significant network assembled de novo using 27 focus molecules associated with selected enriched signalling pathways (mitotic roles of polo-like kinase, AHR signalling, cell cycle regulation by BTG family proteins, cell cycle: G2/M DNA damage checkpoint regulation). The inset shows molecules associated with the selected canonical pathways. **C**. Top significant signalling pathway, mitotic roles of polo-ike kinase, showed that the conserved estrogen-responsive genes encoding the respective molecules were all up-regulated (red nodes) from mitotic entry through transition from metaphase to anaphase and mitotic exit followed by cytokinesis and subsequent centrosome separation. Legends are similar to Figure 3B and 3C.

We used IPA to identify signaling pathways and key biological network enriched by the 139 conserved estrogen-responsive genes. The analysis revealed that signaling pathways involved mainly in cell cycle progression and DNA damage and repair such as mitotic roles of polo-like kinase, CDK5 signaling, cell cycle regulation by BTG family proteins, cell cycle: G2/M DNA damage checkpoint regulation, aryl hydrocarbon receptor (AHR) signaling, ATM signaling and role of CHK proteins in cell cycle checkpoint control were significantly enriched (Fisher's exact test p-value = 1.93E-07 to 3.34E-02; Figure 5A). In order to gain insight into the connectivity of the signaling pathways with key biological functions and genes, we overlay the selected enriched signaling pathways over the most significant network which is associated with DNA replication, recombination and repair, cell cycle and cancer (Figure 5B; Fisher's exact test p-value = 1.00E-43). Among the 27 conserved estrogen-responsive human homologs assembled in the network, 10 of them (37%; *ASF1-B, CDK2, CDC6, CHAF1A, HES1, MCM5, PASK, PLK2, SERPINA1, TIPIN*) have experimentally validated ER binding sites in the human genes providing evidence for ER-mediated transcriptional regulation of these genes.

Interestingly, AHR signaling and mitotic roles of polo-like kinase, shown to be highly connected with different sets of assembled genes/molecules in this network (Figure 5B insert) further confirmed not only their known strong associations with DNA replication, recombination and repair, cell cycle and cancer, but also revealed the potential conserved estrogen-responsiveness of these signaling pathways. AHR signaling is known to demonstrate inhibitory crosstalk with ER signaling [33] and also involved in cell cycle regulation and tumorigenesis [34]. It is interesting to hypothesize from our network analysis that an estrogen-activated ER cum basal AHR crosstalk mechanism may be operating in cell-cycle regulation. Estrogen-activated ER has been reported to compete for recruitment of shared coactivators with AHR for expression of estrogen-responsive genes [35,33]. In addition, estrogen has been reported to stimulate the recruitment of unliganded AHR to the proximal *BRCA-1 *promoter region and potentiate the effects of the liganded ER in activation of *BRCA-1 *transcription [36]. More recently, there has been increasing *in vivo *evidence supporting that *Ahr *gene in its basal state, without any xenobiotic ligand activation, functions as a tumor suppressor gene and that its silencing may be associated with cancer progression [37,38]. Repression or loss of *Ahr *was found to increase expression of proliferative markers and repress tumor suppressor genes leading to increased tumor incidence in rodent models [37-39]. We have also observed down-regulation of basal *cyp1a1 *transcripts, a well-studied *ahr *regulated gene, in multiple tissues of zebrafish exposed to estrogen alone [14] hence suggesting the squelching of basal ahr activity induced by estrogen. Although the nature of basal/unliganded AHR actvities in nucleus are still unclear, nucleocytoplasmic shuttling and nuclear localization of unliganded AHR had been demonstrated in MCF7, murine and zebrafish liver cell lines [40,41]. Based on these evidences, our network analysis suggests that similar mechanism may be operating whereby estrogen stimulates recruitment of basal/unliganded AHR and shared coactivators for transcription regulation of estrogen-responsive genes hence reducing basal AHR tumor suppressing effects. It has been previously proposed that AHR tumor suppressing effects could involve RB-E2F axis since interaction of AHR and RB-E2F protein complex represses G1-S phase transition [38,39]. Our network analysis suggests two potential conserved targets of this ER and AHR signalling crosstalk, i.e. RB-E2F axis and HES1, which are known to be regulated antagonistically by ER and AHR ligands and are central to activating or inhibiting estrogen-mediated cell proliferation [34,42,43].

As captured in the network (Figure 5B), the down-regulation of human homolog *HES1 *and the up-regulation of cell cycle-related genes (e.g. human homologs *E2F4, CDK2, CCNA, CCNE*) provide important evidence of a conserved estrogen-mediated cell cycle regulatory mechanism. HES1, known to be targeted by ER [42,44] and AHR [43], is a transcription repressor that directly represses *E2F1 *and it inhibits cell proliferation[42]. Therefore as represented in the network, the down-regulation of human homolog *HES1 *by estrogen, known to be mediated by a novel type repressive estrogen response element in human [44], appeared to be conserved between fish and human, and this could be pivotal for the up-regulation *E2F *transcription factors and downstream cell cycle genes (human homologs *CDK2, CCNA, CCNE*) that are necessary for G1-S phase transition leading to mitosis. Several *E2F *transcription factors and cell cycle-related genes have been shown to be up-regulated by estrogen as primary or secondary targets in human breast cancer cell lines [45]. Our analysis indicate that *E2F4 *homolog is up-regulated likely to promote cell cycle progression and it has been associated with breast carcinogenesis as previously reported [46,47]. Interestingly, *E2F4 *is also known to repress cell cycle, hence it can exert either tumor-suppressive or oncogenic effects in vivo but the mechanism of this switch is not known [48]. Estrogen-induced up-regulation of human homologs *CDK2*, *CCNE *and *CCNA*, will presumably increase protein complexes such as CDK2/cyclin E and CDK2/cyclin A that partly promote G1-S phase transition and S-G2 phase transition, respectively, before entering M phase [49,50].

A closer examination of a major (and also top significant) signaling pathway in the M phase, mitotic roles of polo-like kinase, showed that the conserved estrogen-responsive genes (human homologs *APC, CCNB1, CCNB2, MKLP1, PLK, PP2A, PRC1*) were all up-regulated (Figure 5C). Among the 7 human homologs, *MKLP1, PLK, PP2A *have experimentally validated ER binding sites providing evidence for a direct ligand activated-ER regulation of this signaling pathway. Hence, through these genes and their encoded products we observed entry points for estrogen regulation of mitosis; from mitotic entry (via PLK, PP2A, CCNB) through transition from metaphase to anaphase and mitotic exit (via PLK, PRC1, CCNB, APC) followed by cytokinesis (via PLK, MKLP1) and subsequent centrosome separation (via PLK, CCNB) (Figure 5C).

Perhaps of greater significance is that our study indicates that estrogen regulation of cell cycle is conserved between actinopterygian fish and humans. Since actinopterygian fish is phylogenetically positioned in the extreme end of the vertebrate taxon when compared to humans, this would suggest that estrogen regulation of cell cycle is likely to be conserved across vertebrates. This is interesting from an evolutionary perspective because estrogen and its receptors are found to be the most ancient steroidal -receptor signalling [51,52]. Given that estrogen modulates many physiological systems in higher vertebrates, it would be interesting to speculate on the ancient physiological process(es) where estrogen regulation of cell cycle is occurring. Although the presence of estrogen and ligand-binding ER outside vertebrate is controversial [53], a recent study in annelid invertebrates [54] has demonstrated that annelid ERs bind estrogen with high affinity and can be activated by low concentration of estrogen. Keay and Thornton [54] postulated that the ancient roles of estrogen and its receptor are involved in regulation of reproductive maturation and function including germ cell maturation and provision of oocyte with vitellogenin. If so, the ancient physiological process where estrogen regulation of cell cycling may be occurring is during germ cell/oocyte maturation. Taken together, our findings would imply that estrogen regulation of cell cycle is likely one of the earliest forms of steroidal-receptor controlled cellular processes within vertebrates and perhaps may even extend back to annelid invertebrates.

## Conclusion

Previously, we have shown that fish and human shared conserved molecular hallmarks of liver tumors and its progression [9]. Based on the transcriptome analysis in this study, we provide evidence that the conservation could possibly extend further to estrogen-induced carcinogenesis since fish is also susceptible to the carcinogens affecting humans. Collectively, our transcriptome analysis captured multiple estrogen-responsive genes and signaling pathways largely responsible for increased cell proliferation, promote DNA damage and genome instability and decreased tumor suppressing effects (including cell death), which suggests a common mechanism for estrogen-induced carcinogenesis. Comparative analysis revealed a core set of conserved estrogen-responsive genes with enriched ER binding sites and a conserved estrogen-mediated cell cycle regulatory mechanism involving HES1, RB-E2F axis and mitotic roles of polo-Like kinase signaling pathway. Almost half of the conserved genes have validated ER binding sites indicating that they are direct ER targets. From an evolutionary perspective, our findings suggest that estrogen regulation of cell cycle is perhaps one of the earliest forms of steroidal-receptor controlled cellular processes. The conserved estrogen-regulated cell cycle signature identified in this study would be useful for characterizing estrogen-like environmental carcinogens and for anti-estrogen compound screening. The findings also warrant further cancer studies in zebrafish to involve estrogen component in experimental design and interpretation of data which would aid in modelling estrogen-responsive cancer in zebrafish. To our knowledge, this is the first report providing evidence for molecular conservation of estrogen responsiveness between zebrafish and human cancer cell lines, hence demonstrating the potential of zebrafish for estrogen-related cancer research.

## Abbreviations

AhR: Aryl hydrocarbon receptor; ARNT: Aryl hydrocarbon receptor nuclear translocator; Aro-B: brain aromatase; ASF1B: ASF1 anti-silencing function 1 homolog B; BHLHB2: Basic helix-loophelix domain containing class B 2; *CCNA*: Cyclin A; CCNA1: Cyclin A1; CCNB: Cyclin B; CCNB1: Cyclin B1; CCNB2: Cyclin B2; CCNE: Cyclin E; CCNE2: Cyclin E2; CCNG2: Cyclin G2; CDC6: Cell division cycle 6 homolog; CDK2: Cyclin-dependent kinase 2; ChIP: Chromatin Immuno-Precipitation; E2,17β-estradiol; E2F: E2F transcription factor; E2F4: E2F transcription factor 4; EE2: 17-alpha-ethynylestradioll; EGR1: Early growth response 1; ELF3: E74-like factor 3; ERE: estrogen-response element; ERK: extracellular signal-regulated kinases; ERα: Estrogen receptor alpha; ERβ: Estrogen receptor beta; esr1: zebrafish homolog of estrogen receptor alpha; ESTs: Expressed sequenc tags; FOXA1: Forkhead box A1; HES1: Hairy and enhancer of split 1; ICI: ICI 182,780; IPA: Ingenuity Pathways Analysis; JUN: Jun oncogene; MAPK: Map kinase(mitogen-activated protein kinase); MCM: Minichromosome maintenance complex; MCM3: Minichromosome maintenance complex component 3; MCM5: Minichromosome maintenance complex component 5; MCM6: Minichromosome maintenance complex component 6; OLFM1: Olfactomedin 1; PCNA: Proliferating cell nuclear antigen; PPP2R1B: Protein phosphatase 2 (formerly 2A) regulatory subunit A: beta isoform; SAM: Significance Analysis of Microarrays; SIAH1,Seven in absentia homolog 1 (Drosophila); *Sox11b*: SRY(sex determining region Y)-box containing gene 11b; sox19a: SRY(sex determining region Y)-box containing gene 19a; sox19b: SRY(sex determining region Y)-box containing gene 19b; sox21: SRY(sex determining region Y)-box containing gene 21; sox31: SRY(sex determining region Y)-box containing gene 31a; TOP2A: Topoisomerase (DNA) II alpha; UCSC: University of California Santa Cruz; vtg1: vitellogenin 1; vtg3: vitellogenin 3; ZGC: Zebrafish Gene Collection; zp1: Zona pellucida glycoprotein 1; zp3: Zona pellucida glycoprotein 3.

## Competing interests

The authors declare that they have no competing interests.

## Authors' contributions

SM, ZG and ETL conceived and designed the experiments; SHL, SL, SM performed the experiments, CYL, and PYF generated part of the data for human cells and critically read the paper, SHL and SM performed IPA analysis, CHL and GB analyzed ER binding site and ERE motif search, KRK, HL and KRG analyzed the data and generated annotation tools. SHL and SM wrote the paper and TL, ZG, CYL and ETL significantly contributed in finalizing the manuscript. All authors read and approved the final manuscript.

## Pre-publication history

The pre-publication history for this paper can be accessed here:

http://www.biomedcentral.com/1755-8794/4/41/prepub

## Supplementary Material

Additional file 1 **qRT-PCR primers**. Contains the list of genes and their primers used for the validation of the microarray data representing the up and down regulated genes. Click here for file

Additional file 2 **E2 responsive genes from MCF7 cells**. List of estrogen responsive genes identified from MCF7 cells by Carroll et al [21] are presented in this file. Click here for file

Additional file 3 **E2 responsive genes from T47D cells**. List of estrogen responsive genes identified from T47D cells by Lin et al [20] are presented in this file. Click here for file

Additional file 4 **E2 responsive genes from MCF7 cells**. List of estrogen responsive genes identified in MCF7 cells by Thomsen et al (2008; unpublished) are presented in this file. Click here for file

Additional file 5 **E2 responsive genes from Ishikawa cells**. List of estrogen responsive genes identified from Ishiwaka cells by Thomsen et al (2008; unpublished) are presented in this file. Click here for file

Additional file 6 **E2 responsive gene in male zebrafish (Up-regulated genes)**. Zebrafish genes significantly up-regulated due to estrogen and estrogen+ICI treatments are presented in this file. Click here for file

Additional file 7 **E2 responsive gene in male zebrafish (down-regulated genes)**. Zebrafish genes significantly down-regulated due to estrogen and estrogen+ICI treatments are presented in this file. Click here for file

Additional file 8 **Annotation E2 responsive genes of zebrafish based on UniGene clusters**. Estrogen responsive genes identified for zebrafish were annotated and grouped as (i) known genes, (ii) ZGC clone and (iii). different categories of ESTs. A. Annotation of up-regulated genes. B. Annotation of down-regulated genes. A number of putative target genes for estrogen have been identified from the annotated dataset. Click here for file

Additional file 9 **Human homolog of E2 responsive zebrafish genes**. Zebrafish E2 responsive genes were mapped to homologene data base and identified human homologs are presented in this file. Click here for file

Additional file 10 **Expression patterns of subset of E2 responsive zebrafish specific genes**: Description: Of the estrogen responsive zebrafish genes, a subset of data (ZGC clones) is included here. These genes are significantly up-regulated in E2 treated male fish and their response to estrogen has been discovered for the first time. These genes appear to be fish specific and do not have human homolog. Click here for file

Additional file 11 **IPA analysis of E2 responsive genes**. Top 10 enriched functional groups in the E2 responsive genes identified by IPA are presented in this file. Click here for file

Additional file 12 **E2 responsive zebrafish genes associated with cancer**: Human homologs of zebrafish E2 responsive genes associated with different cancer types are presented in this file. Click here for file

Additional file 13 **IPA generated gene networks**: Top five gene network generated by E2 responsive genes and the molecules involved in the net work are presented in this list. Click here for file

Additional file 14 **Conserved E2 responsive genes between zebrafish and human cell lines**. 139 Conserved Estrogen Responsive Genes between Zebrafish and Human Cancer Cell Lines with or without ER binding sites. Click here for file

Additional file 15 **E2 responsive overlapping genes between zebrafish and human cell lines**. Estrogen responsive genes identified in zebrafish and each human cell lines were compared using Venn diagram. Numbers of overlapping genes and the percentage of overlap are represented in each Venn diagram. Click here for file

Additional file 16 **E2 responsive overlapping genes between human cell lines**. Estrogen responsive genes identified in human cells were compared using Venn diagram. Numbers of overlapping genes and the percentage of overlap between each cell line is presented. Overall overlap between these cell lines are presented in a four group Venn diagram. Click here for file

Additional file 17 **ERE motif analysis**. A. Distribution of ERE motif: Distribution of ERE motif (GGTCAnnnTGACC) in the flanking regions (20 Kb in 5' region; 20Kb in 3' region) from the TSS in the estrogen responsive genes of zebrafish and in the whole genome was determined and presented. The distribution of ERE motif appears to be random without specific enrichment in the flanking regions of estrogen responsive genes. B. ER binding site analysis in the human homologs of E2 responsive zebrafish genes: The analysis was done in the proximity of 100kb of these genes. We observe an enrichment of ER binding site in the conserved genes (E2 responsive both in zebrafish and human cancer cells) compared to non-overlapping gens. Randomisation was done by randomly selecting the same number of genes from the entire KnowGene collection and mapping to the same ER binding sites over 1000 times. Click here for file
